# Sex differences in clinical risk factors in obese ischemic stroke patients with a history of smoking

**DOI:** 10.1186/s12872-024-03952-6

**Published:** 2024-05-30

**Authors:** Dami T. Ojo, Philip C. Brewer, Adebobola Imeh-Nathaniel, Samuel Imeh-Nathaniel, Philip X. Broughton, Thomas I. Nathaniel

**Affiliations:** 1 University of South Carolina School of Medicine-Greenville, Greenville, SC 29605 USA; 2 North Greenville University Tigerville, Tigerville, SC USA

**Keywords:** Acute ischemic stroke, Obesity, Smoking, Males, Females

## Abstract

**Abstract:**

Clinical risk factors associated obesity and smoking, as well as their combined effect, are not fully understood. This study aims to determine sex differences in risk factors in a population of acute ischemic stroke (AIS) patients who are obese and with a history of previous or current smoking.

**Methods:**

A retrospective analysis of risk factors in male and female AIS patients with baseline data of obesity and current or previous history of smoking, smoking, and obesity alone was determined. The primary predictor and outcome are risk factors associated with male and female AIS patients. Baseline risk factors were analyzed using a multivariate regression analysis to determine specific risk factors linked with the combined effect of obesity and current or previous history of smoking’’.

**Results:**

Male obese AIS patients who are current or previous smokers were more likely to be older patients(OR = 1.024, 95% CI, 1.022–1.047, *P =* 0.033) that present with coronary artery disease (OR = 1.806, 95% CI, 1.028–3.174, *P* = 0.040), a history of alcohol use (OR = 2.873, 95% CI, 1.349–6.166, *P* = 0.006), elevated serum creatinine (OR = 4.724, 95% CI, 2.171–10.281, *P <* 0.001) and systolic blood pressure (OR = 1.029, 95% CI, 1.011–1.047, *P <* 0.002). Females were more associated with depression (OR = 0.432, 95% CI, 0.244–0.764, *P =* 0.004), previous TIA (OR = 0.319, 95% CI, 0.142–0.714, *P <* 0.005), and higher levels of HDL (OR = 0.938, 95% CI, 0.915–0.962, *P <* 0.001).

**Conclusion:**

Our results reveal sex differences in risk factors in obese AIS patients with a current or past history of smoking. This finding emphasizes the need to develop management strategies to improve the care of obese AIS patients who are either current or former smokers.

## Introduction

Despite substantial progress in research focusing on sex differences in comorbidities influencing outcomes in male and female post-stroke patients who smoke [1–3], there are still major research gaps in stroke risk factors specific for male and female smokers and obese individuals. While several studies have shown that smoking is not associated with an increase in body weight [4], smoking is known to be associated with central obesity [5, 6]. Interestingly, only males that are former smokers were reported to be at risk of central obesity [5]. Obesity and smoking have each been linked to cerebrovascular diseases including stroke [7, 8]. In addition, obese individuals who are smokers present with a risk of circulatory and cerebrovascular diseases with increased mortality by 7- to 10-fold for smokers compared to normal-weight and non-smokers [9]. Both smoking and obesity have been linked to increased incidence of stroke in both male and female AIS patients [10, 11], but sex differences in obese AIS patients with a current or previous history of smoking are not fully understood.

While stroke ranks as the fourth leading cause of death among individuals aged 20 to 59, it emerges as the second leading cause of death in females aged 60 and over [12, 13]. Among those aged 45–74, males exhibit a higher rate of stroke and mortality. However, beyond the age of 74, females tend to surpass males in both stroke incidence and mortality [14, 15]. Females often present with worse baseline function, more comorbidities, poorer post-stroke outcomes, and an overall greater risk for stroke compared with males [16, 17]. In addition, stroke severities, and deaths linked to a stroke are less in males than females [18]. The higher severity and death rate in females is linked to the older age of females at stroke onset and the fact that females present with longer life expectancy compared with males [19, 20].

Prospective studies provided evidence for the effects of risk factors on the risk of stroke, and most of the risk factors are at the disadvantage of females [21, 22]. For example, diabetes causes more risk of stroke in females than in males [23, 24]. Similarly, a history of atrial fibrillation was associated with a higher risk of stroke in females compared with males [21, 25]. In terms of smoking and obesity, more than 25% of males and 6% of females are reported to smoke every day [26, 27], and smoking alone or in combination with obesity poses a major risk for stroke for both males and females compared with non-obese or smoking individuals [28, 29]. While obesity is reported to be more prevalent in females than males [30, 31], males tend to smoke at higher rates than females [32, 33].

Given that there is a stronger association between more risk factors and the severity of stroke in females compared with males [34], it is possible that risk factors for ischemic stroke in individuals with a combination of obesity and smoking may not be equally distributed between females and males. One possibility is that obese females may present with more risk factors when compared with obese male AIS patients with a current or previous history of smoking. Therefore, the first objective of this study is to identify the risk factors in obese male and female AIS patients with a current or previous history of smoking.

Since obese males and females with a history of smoking may not present with similar risk factors in the general AIS population, our second objective is to determine specific risk factors contributing to sex differences in obese AIS patients with a history of smoking. Understanding these sex-related risk factors can facilitate the development of targeted support and personalized care to manage risk factors in obese AIS patients with a current or previous history of smoking.

## Methods

### Study population

This retrospective study utilized data obtained from the Prisma Health Acute Ischemic Stroke (AIS) cohort. The study protocol was reviewed and approved by the institutional review board of the PRISMA Health Institutional Committee for Ethics. Brain imaging (MRI, MRA, CT, CTA, etc.) was used to validate cases of ischemic stroke and to exclude cases with intracerebral or subarachnoid hemorrhagic stroke. We used data for patients classified as obese based on the BMI guidelines established by the World Health Organization (WHO) with individuals with a BMI of (> 30) being classified as obese and individuals with a BMI (< 30) falling into the categories of underweight, normal weight, or overweight [35]. Patients were classified as smokers based on whether the history of smoking was current, i.e. immediately before stroke or previous history before stroke. Therefore, smokers in this study are those with a current or previous history of smoking before admission for treatment of ischemic stroke. Data on clinical variables, demographic factors, and comorbidities were obtained from the registry.

Demographic factors including gender, age, ethnicity, and race were considered in this study. Additionally, past medical history of the patient population was also obtained covering conditions such as atrial fibrillation, coronary artery disease (CAD), carotid stenosis, depression, diabetes, a history of substance abuse, hypertension, migraine, heart failure (HF), hormone replacement therapy (HRT), dyslipidemia, family history of stroke, obesity, prior stroke, prior TIA, prosthetic heart valve, peripheral vascular disease (PVD), chronic renal disease, sickle cell, and sleep apnea. Ambulatory data from the patients were also collected with scores ranging from 0 to 3 0 denoting undocumented ambulation ability, 1 indicating an inability to ambulate, 2 denoting ability to ambulate with assistance, and 3 denoting the ability to ambulate independently. These scores were assigned to patients before, during, and after discharge. Any improvement was documented and defined as an increase in ambulatory status from patient admission to discharge.

### Statistical analysis

Statistical Analyses were performed using Statistical Package for Social Sciences version 29.0 for Mac (SPSS, Chicago, IL). The normality of data distribution was assessed using the Kolmogorov-Smirnov test. Continuous variables with normal distribution were presented as mean ± SD, and comparisons between male and female patients were determined using the student-t-test. Non-normally distributed variables were analyzed using the Mann‐Whitney U test. Categorical variables were described using frequencies, and group differences were assessed using the χ^2^ test or Student T-Test. Patients were categorized into three groups (smokers, obesity, and smokers and obese), and univariate analysis was used to determine sex differences in risk factors for each group. The power analysis was computed using PASS 2023, version 23.0.2. For 2807 females, the power was 91.23% and d = 0.24, while 2662 males produced a power of 90% and d = 0.20.

The primary analysis in our study was sex-associated risk factors in male and female patients as outcome, performed using logistic regression models (with medical history, and laboratory values. as predictors in all models). Models were developed for each of the 4 AIS groups including,1) the whole AIS population, 2) smoking, 3) obesity, and 4) smoking combined with obesity stroke population. Our logistic regression models identified risk factors associated with male or female AIS patients within each of the stroke groups, including variables such as smoking, obesity, and the combined effects of obesity with a current or previous history of smoking. We assessed multicollinearity using Hosmer-Lemeshow tests while determining the sensitivity and specificity of the models through overall classified percentage and the area under the receiver operating curve (AUROC). Odds Ratios (ORs) for gender-specific risk factors in male and females were determined at 95% confidence intervals (CIs). The significance level for all tests was set at *P* < 0.05.

## Results

A total of 5,469 individuals diagnosed with ischemic stroke, were identified. Of this, 2,662 were males and 2,807 were females (Table 1). Male patients were more likely to present with a history of coronary artery disease, carotid artery stenosis, alcohol use, sleep apnea, and tobacco use (*P* < 0.001). Conversely, females presented with a higher likelihood of presenting with atrial fibrillation, depression, heart failure, hypertension, and migraines (*P* < 0.05). Males were more likely to be treated with cholesterol reducers (*P* < 0.05 while females were more likely to be treated with antihypertensive medications and antidepressants (*P* < 0.05). Females were likely to be older, and present with higher BMI compared with males (*P* < 0.05). Males were likely to present with higher levels of serum creatinine (*P* < 0.05), while females were likely to present with higher total cholesterol, HDL, and LDL (*P* < 0.05). Vital signs including higher diastolic blood pressure were more likely to be associated with males(*P* < 0.05). Females presented with a higher incidence of heart rate (*P* < 0.05) while males presented with improved ambulation (*P* < 0.05) when compared with females.

**Table 1:** Demographic and clinical characteristics of ischemic stroke patients divided by sex

Characteristic	Male	Female	
**Number of patients**	2,662	2,807	***P-*** **value**
**Age Group: No. (%)**			
< 50	305 (11.5)	353 (12.6)	< 0.001*^a^
50–59	603 (22.7)	393 (14.0)	
60–69	740 (27.8)	559 (19.9)	
70–79	592 (22.2)	639 (22.8)	
>=80	422 (15.9)	30.7)	
**Age Mean ± SD**	65.13 ± 13.372	69.26 ± 15.657	< 0.001*^b^
Race: No (%)			
White	2,090 (78.5)	2,198 (78.3)	0.544
Black	492 (18.5)	510 (18.2)	
Other	80 (3.0)	99 (3.5)	
Hispanic Ethnicity: No. (%)	38 (1.4)	47 (1.7)	0.461
BMI: Mean ± SD	28.04 ± 5.983	28.61 ± 7.795	0.002*^b^
**Medical History: No. (%)**			
Atrial Fib	381 (14.3)	543 (19.3)	< 0.001*^a^
Coronary Artery Disease	935 (35.1)	726 (25.9)	< 0.001*^a^
Carotid Artery Stenosis	191 (7.2)	143 (5.1)	0.001*^a^
Depression	271 (10.2)	450 (16.0)	< 0.001*^a^
Diabetes	951 (35.7)	984 (35.1)	0.605
Drugs or Alcohol	256 (9.6)	81 (2.9)	< 0.001*^a^
Dyslipidemia	1,364 (51.2)	1,391 (49.6)	0.213
Stroke Family History	224 (8.4)	270 (9.6)	0.121
Heart Failure	245 (9.2)	345 (12.3)	< 0.001*^a^
Hypertension	2,056 (77.2)	2,250 (80.2)	0.008*^a^
Migraine	29 (1.1)	105 (3.7)	< 0.001*^a^
Obesity	1,148 (43.1)	1,163 (41.4)	0.205
Previous Stroke	671 (25.2)	753 (26.8)	0.173
Previous TIA (> 24 h)	213 (8.0)	264 (9.4)	0.066
Prosthetic Heart Valve	35 (1.3)	27 (1.0)	0.218
Peripheral Vascular Disease	192 (7.2)	208 (7.4)	0.779
Chronic Renal Disease	232 (8.7)	215 (7.7)	0.154
Sleep Apnea	101 (3.8)	69 (2.5)	0.004*^a^
Smoker	883 (33.2)	603 (21.5)	< 0.001*^a^
**Medication History: No (%)**			
HTN medication	1,763 (66.2)	2,031 (72.4)	< 0.001*^a^
Cholesterol Reducer	1,227 (46.1)	1,201 (42.8)	0.014*^a^
Diabetic Medication	735 (27.6)	760 (27.1)	0.657
Antidepressant	251 (9.4)	460 (16.4)	< 0.001*^a^
**Initial NIHSS Score: No (%)**			
0–9	1,677 (74.0)	1,612 (69.5)	0.005*^a^
10–14	235 (10.4)	272 (11.7)	
15–20	218 (9.6)	283 (12.2)	
21–25	137 (6.0)	153 (6.6)	
Mean ± SD	7.63 ± 7.84	8.90 ± 8.56	< 0.001*^b^
**Lab values: Mean ± SD**			
Total cholesterol	165.96 ± 52.10	177.54 ± 50.89	< 0.001*^b^
Triglycerides	142.60 ± 110.95	136.78 ± 99.16	0.057
HDL	38.43 ± 12.57	45.03 ± 14.23	< 0.001*^b^
LDL	101.83 ± 39.72	107.31 ± 42.58	< 0.001*^b^
Lipids	6.51 ± 1.82	6.54 ± 3.11	0.709
Blood Glucose	146.75 ± 78.26	147.82 ± 83.62	0.626
Serum Creatinine	1.40 ± 1.22	1.18 ± 1.09	< 0.001*^b^
INR	1.15 ± 0.51	1.18 ± 1.09	0.069
**Vital Signs: Mean ± SD**			
Heart Rate	80.43 ± 15.35	83.50 ± 18.57	< 0.001*^b^
Blood Pressure Systolic	152.09 ± 28.86	151.57 ± 29.74	0.508
Blood Pressure Diastolic	84.60 ± 18.37	80.39 ± 19.59	< 0.001*^b^
**Ambulation Status Prior to Event: No. (%)**			
Ambulate Independently	2,453 (92.1)	2,434 (86.7)	< 0.001*^a^
Ambulate with Assistance	74 (2.8)	129 (4.6)	
Unable to Ambulate	79 (3.0)	134 (4.8)	
Not Documented	56 (2.1)	109 (3.9)	
**Ambulation Status on Admission: No. (%)**			
Ambulate Independently	727 (27.3)	604 (21.5)	< 0.001*^a^
Ambulate with Assistance	790 (29.7)	836 (29.8)	
Unable to Ambulate	744 (27.9)	984 (35.1)	
Not Documented	401 (15.1)	383 (13.6)	
**Ambulation Status on Discharge: No. (%)**			
Ambulate Independently	1,184 (44.5)	990 (35.3)	< 0.001*^a^
Ambulate with Assistance	846 (31.8)	974 (34.7)	
Unable to Ambulate	429 (16.1)	640 (22.8)	
Not Documented	203 (7.6)	203 (7.2)	
rtPA received: No. (%)	668 (25.1)	659 (23.5)	0.163
**First Care Received: No. (%)**			
Emergency Department	2,085 (79.1)	2,212 (79.4)	0.806
Direct Admission	550 (20.9)	574 (20.6)	
Improved Ambulation: No. (%)	927 (37.5)	898 (34.4)	0.019*^a^
NIHSS > 7: No. (%)	824 (35.0)	1,043 (42.1)	< 0.001*^a^
Diastolic Blood Pressure ≥ 80 mmHg	1,576 (59.3)	1,312 (46.8)	< 0.001*^a^

Results for continuous variables are presented as Mean ± SD, while discrete data are presented as percentage frequency. Pearson’s Chi-Square is used to compare differences between demographic and clinical characteristics in male and female ischemic stroke patients

^a^Pearson’s Chi-Squared test

^b^Student’s T test

* *P*-value < 0.05

Table 2 presents the risk factors of AIS patients stratified by sex and obesity with current or previous history of smoking, obesity, and smoking alone. Obese AIS with a history of smoking that are males were more likely to present with coronary artery disease and alcohol use (*P* < 0.05). However, females were more likely to present with depression and migraines and were more likely to be treated with diabetic medications and antidepressants (*P* < 0.05). Males were more likely to present with higher serum creatinine levels (*P* < 0.05), while females were more likely to present with higher total cholesterol, and HDL levels (*P* < 0.05). Systolic and diastolic blood pressure were higher in males (*P* < 0.05 )when compared with females in the obese AIS patients with a history of smoking.

**Table 2:** Demographic and clinical characteristics between male and female ischemic stroke patients stratified by smoking and obesity (smoking + obesity, smoking, and obesity)

	Smoking + Obesity			Smoking				Obesity			
Characteristic	Male	Female		Male	Female		Male		Female		
Number of patients	354	254	P-value	883	603	p-value		1148	1163	p-value	
**Age Group: No. (%)**											
< 50 years	75 (21.2)	67 (26.4)	0.600	160 (18.1)	148 (24.5)	0.006* ^a^		151 (13.2)	185 (15.9)	< 0.001* ^a^	
50–59	140 (39.5)	95 (37.4)		313 (35.4)	179 (29.7)			294 (25.6)	203 (17.5)		
60–69	102 (28.8)	64 (25.2)		286 (32.4)	173 (28.7)			313 (27.3)	265 (22.8)		
70–79	32 (9.0)	25 (9.8)		100 (11.3)	84 (13.9)			249 (21.7)	256 (22.0)		
>=80	5 (1.4)	3 (1.2)		24 (2.7)	19 (3.2)			141 (12.3)	254 (21.8)		
Age Mean ± SD	57.02 ± 10.348	65.80 ± 12.333	0.199	58.51 ± 10.888	57.80 ± 12.839	0.267		63.68 ± 13.139	66.07 ± 15.412	< 0.001* ^b^	
**Race: No (%)**											
White	255 (72.0)	188 (74.0)	0.196	633 (71.7)	448 (74.3)	0.540		905 (78.8)	887 (76.3)	0.023*^a^	
Black	97 (27.4)	61 (24.0)		214 (24.2)	113 (22.1)			234 (20.4)	252 (21.7)		
Other	2 (0.6)	5 (2.0)		36 (4.1)	22 (3.6)			9 (0.8)	24 (2.1)		
Hispanic Ethnicity: No. (%)	1 (0.3)	3 (1.2)	0.176	8 (0.9)	5 (0.8)	0.876		18 (1.6)	26 (2.2)	0.240	
BMI: Mean ± SD	32.1718 ± 7.10477	30.8865 ± 5.17968	0.613	27.3098 ± 5.9962	27.8316 ± 7.49845	0.157		30.9605 ± 5.135	32.7794 ± 7.00792	< 0.001* ^b^	
**Medical History: No. (%)**											
Atrial Fib	22 (6.2)	11 (4.3)	0.206	49 (5.5)	36 (6.0)	0.732		166 (14.5)	171 (14.7)	0.868	
Coronary Artery Disease	100 (28.2)	57 (22.4)	0.029* ^a^	230 (26.0)	121 (20.1)	0.008* ^a^		398 (34.7)	304 (26.1)	< 0.001*^a^	
Carotid Artery Stenosis	17 (4.8)	20 (7.9)	0.229	62 (7.0)	42 (7.0)	0.967		70 (6.1)	59 (5.1)	0.283	
Depression	56 (15.8)	68 (26.8)	< 0.001* ^a^	78 (8.8)	102 (16.9)	< 0.001* ^a^		187 (16.3)	311 (26.7)	< 0.001*^a^	
Diabetes	112 (31.6)	94 (37.0)	0.278	224 (25.4)	184 (30.5)	0.029 * ^a^		484 (42.2)	502 (43.2)	0.626	
Drugs or Alcohol	85 (24.0)	29 (11.4)	0.015* ^a^	161 (18.2)	60 (10.0)	< 0.001* ^a^		145 (12.6)	39 (3.4)	< 0.001*^a^	
Dyslipidemia	162 (45.8)	122 (48.0)	0.145	350 (39.6)	263 (43.6)	0.126		645 (56.2)	632 (54.3)	0.373	
Stroke Family History	52 (14.7)	42 (16.5)	0.291	82 (9.3)	68 (11.3)	0.211		151 (13.2)	174 (15.0)	0.211	
Heart Failure	30 (8.5)	20 (7.9)	0.623	62 (7.0)	44 (7.3)	0.840		103 (9.0)	145 (12.5)	0.007	
Hypertension	267 (75.4)	200 (78.7)	0.826	639 (72.4)	443 (73.5)	0.640		925 (80.6)	959 (82.5)	0.243	
Migraine	9 (2.5)	16 (6.3)	0.003* ^a^	12 (1.4)	27 (4.5)	< 0.001* ^a^		22 (1.9)	75 (6.4)	< 0.001*^a^	
Obesity	354 (100)	254 (100)		354 (40.1)	254 (42.1)	0.434		1148 (100)	1163 (100)		
Previous Stroke	95 (26.8)	58 (22.8)	0.323	235 (26.6)	161 (26.7)	0.971		283 (24.7)	305 (26.2)	0.385	
Previous TIA (> 24 h)	19 (5.4)	29 (11.4)	0.051	48 (5.4)	60 (10.0)	< 0.001* ^a^		92 (8.0)	100 (8.6)	0.611	
Prosthetic Heart Valve	6 (1.7)	2 (0.8)	0.149	5 (0.8)	7 (0.8)	0.939		20 (1.7)	14 (1.2)	0.282	
Peripheral Vascular Disease	33 (9.3)	29 (11.4)	0.979	59 (6.7)	46 (7.6)	0.484		95 (8.3)	101 (8.7)	0.724	
Chronic Renal Disease	37 (10.5)	18 (7.1)	0.708	61 (6.9)	32 (5.3)	0.211		146 (12.7)	144 (12.4)	0.807	
Smoker	354 (100)	254 (100)		883 (100)	603 (100)			354 (30.8)	254 (21.8)	< 0.001*	
**Medication History: No (%)**											
HTN medication	214 (60.5)	161 (63.4)	0.053	488 (55.3)	365 (60.5)	0.044		794 (69.2)	856 (73.6)	0.018*^a^	
Cholesterol Reducer	132 (37.3)	106 (41.7)	0.668	308 (34.9)	227 (37.6)	0.276		571 (49.7)	557 (47.9)	0.375	
Diabetic Medication	86 (24.3)	79 (31.1)	0.005* ^a^	152 (17.2)	147 (24.4)	< 0.001* ^a^		381 (33.2)	401 (34.5)	0.512	
Antidepressant	47 (13.3)	58 (22.8)	< 0.001* ^a^	62 (7.0)	88 (14.6)	< 0.001* ^a^		175 (15.2)	330 (28.4)	< 0.001*^a^	
**Lab values: Mean ± SD**											
Total cholesterol	181.53 ± 76.327	187.11 ± 51.082	0.004* ^b^	174.22 ± 60.033	183.32 ± 54.599	0.005* ^b^		171.37 ± 59.334	181.46 ± 50.294	< 0.001*^b^	
Triglycerides	177.2 ± 115.08	192.64 ± 164.952	0.237	151.77 ± 111.341	164.73 ± 138.961	0.062		159.68 ± 108.100	155.09 ± 107.128	0.337	
HDL	34.17 ± 10.417	39.04 ± 12.102	0.001* ^b^	37.88 ± 13.541	41.73 ± 13.162	< 0.001* ^b^		35.80 ± 10.721	42.39 ± 12.633	< 0.001*^b^	
LDL	115.0 ± 38.650	116.47 ± 42.469	0.143	108.66 ± 38.973	112.14 ± 43.503	0.133		107.51 ± 40.631	111.55 ± 41.945	0.027*^b^	
Lipids	6.489 ± 1.878	6.5703 ± 1.938	0.100	6.3014 ± 1.74380	6.6422 ± 4.132567	0.080		6.7168 ± 1.94715	6.7079 ± 1.96249	0.919	
Blood Glucose	146.3 ± 81.823	150.45 ± 94.296	0.116	137.34 ± 73.964	144.53 ± 92.177	0.113		155.25 ± 85.430	157.26 ± 93.153	0.589	
Serum Creatinine	1.325 ± 0.99341	1.0706 ± 0.63563	< 0.001* ^b^	1.2878 ± 0.96504	1.0387 ± 0.86639	< 0.001* ^b^		1.4087 ± 1.8028	1.2303 ± 1.10884	< 0.001*^b^	
INR	1.063 ± 0.16202	1.0318 ± 0.11814	0.095	1.0930 ± 0.40391	1.0466 ± 0.19582	0.021		1.1190 ± 0.28165	1.1021 ± 0.35308	0.254	
**Vital Signs: Mean ± SD**											
Heart Rate	82.97 ± 18.333	83.44 ± 16.055	0.179	81.42 ± 18.212	82.65 ± 17.310	0.190		81.06 ± 18.099	83.96 ± 17.866	< 0.001*^b^	
Blood Pressure Systolic	153.1 ± 30.340	148.54 ± 29.401	0.036*^a^	152.96 ± 30.223	148.49 ± 30.081	0.005*^a^		152.12 ± 28.638	151.28 ± 29.457	0.451	
Blood Pressure Diastolic	89.11 ± 20.691	81.80 ± 18.692	< 0.001*^a^	87.93 ± 19.254	81.47 ± 18.845	< 0.001*^a^		84.80 ± 18.256	80.54 ± 19.602	< 0.001*^b^	
**Ambulation Status Prior to Event: No. (%)**											
Ambulate Independently	340 (96)	244 (96.1)	0.589	836 (94.7)	568 (4.2)	0.690		1078 (93.9)	1049 (90.2)	< 0.001*^a^	
Ambulate with Assistance	5 (1.4)	1 (0.4)	0.875	18 (2.0)	9 (1.5)	0.439		25 (2.2)	28 (2.4)	0.712	
Unable to Ambulate	4 (1.1)	3 (1.2)	0.231	14 (1.6)	14 (2.3)	0.305		25 (2.2)	47 (4.0)	0.010*^a^	
Not Documented	5 (1.4)	6 (2.4)	0.853	15 (1.7)	12 (2.0)	0.680		20 (1.7)	38 (3.3)	0.019*^a^	
**Ambulation Status on Admission: No. (%)**											
Ambulate Independently	129 (36.4)	81 (31.9)	0.757	268 (30.4)	176 (29.2)	0.630		379 (33.0)	306 (26.3)	0.001*^a^	
Ambulate with Assistance	80 (22.6)	66 (26.0)	0.689	255 (28.9)	186 (30.8)	0.415		281 (24.5)	291 (25.0)	0.762	
Unable to Ambulate	84 (23.7)	65 (25.6)	0.927	225 (25.5)	159 (26.4)	0.701		287 (25.0)	361 (31.0)	0.001*^a^	
Not Documented	61 (17.2)	42 (16.5)	0.276	135 (15.3)	82 (13.6)	0.365		201 (17.5)	205 (17.6)	0.941	
**Ambulation Status on Discharge: No. (%)**											
Ambulate Independently	219 (61.9)	128 (50.4)	0.739	460 (52.1)	283 (46.9)	0.051		610 (53.1)	509 (43.8)	< 0.001*^a^	
Ambulate with Assistance	82 (23.2)	76 (29.9)	0.491	264 (29.9)	204 (33.8)	0.109		296 (25.8)	362 (31.1)	0.004*^a^	
Unable to Ambulate	40 (11.3)	40 (15.7)	0.697	105 (11.9)	86 (14.3)	0.180		172 (15.0)	234 (20.1)	0.001*^a^	
Not Documented	13 (3.7)	10 (3.9)	0.249	54 (6.1)	30 (5.0)	0.350		70 (6.1)	58 (5.0)	0.243	
rtPA Administration	112 (31.6)	75 (29.5)	0.883	234 (26.5)	154 (25.5)	0.679		341 (29.7)	337 (29.0)	0.701	
**First Care Received: No. (%)**											
Emergency Department	279 (78.8)	185 (72.8)	0.428	684 (77.5)	444 (73.6)	0.090		937 (81.6)	918 (78.9)	0.105	
Direct Admission	38 (10.7)	35 (13.8)	0.107	61 (6.9)	49 (8.1)	0.401		106 (9.2)	136 (11.7)	0.052	
Improved Ambulation: No (%)	147 (43.0)	85 (34.8)	0.198	341 (41.0)	201 (35.0)	0.024*^a^		436 (40.2)	424 (38.2)	0.340	

*Notes*

Results for continuous variables are presented as Mean ± SD, while discrete data are presented as percentage frequency. Pearson’s Chi-Square is used to compare differences between demographic and clinical characteristics between genders

^a^Pearson’s Chi-Squared test

^b^Student’s T test

* P-value < 0.05

Obese male AIS patients with a history of smoking were more likely to present with coronary artery disease and alcohol use *P* < 0.05). On the other hand, females were more likely to present with depression, diabetes, migraine, and previous TIA (*P* < 0.05).

In addition, females were more likely to take hypertensive medications, diabetic medications, and antidepressants, presenting with a higher total cholesterol level and HDL (*P* < 0.05). Males were more likely to present with a higher serum creatinine level, INR, systolic, and diastolic blood pressure and were more likely to present with improved ambulation (*P* < 0.05) when compared with females.

Obese female AIS patients were more likely to be older and presented with higher BMI when compared with males (*P* < 0.05). Males were more likely to present with CAD, and a history of alcohol and tobacco use (*P* < 0.05) while females were more likely to present with higher levels of depression, heart failure, and migraine (*P* < 0.05). Females were more likely to present with higher total cholesterol, HDL, and LDL (*P* < 0.05). Males were more likely to present with higher serum creatinine (*P* < 0.05) while females were more likely to be taking hypertensive medications and antidepressants (*P* < 0.05).

**Fig. 1:**
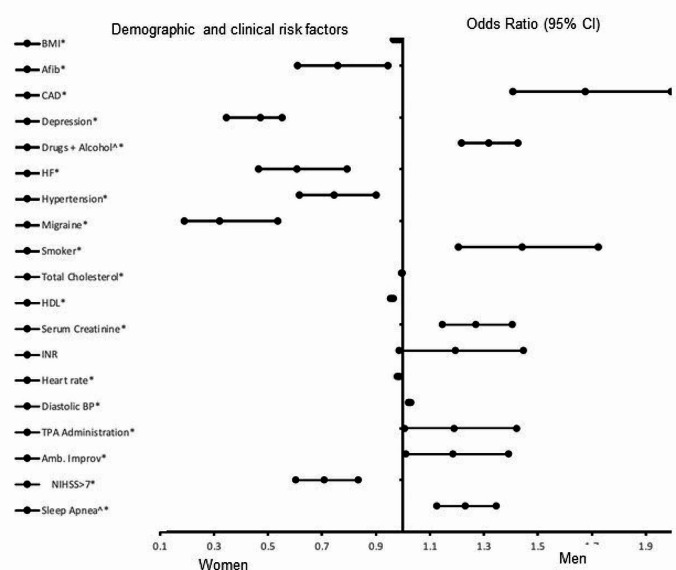
Risk factors associated with male and female AIS patients for the entire AIS population include smoking + obesity, smoking, and obesity. Odd ratios (OD) below 1 denote factors associated with female while above 1 denotes factors associated with male patients. *Indicates statistical significance (*P* < 0.05) with a 95% confidence interval

Figure 1 presents the results of the adjusted analysis for the entire AIS population. BMI (OR = 0.977, 95% CI, 0.966–0.988, P = < 0.001), atrial fibrillation (OR = 0.759, 95% CI, 0.610–0.945, *P* = 0.014), depression (OR = 0.473, 95% CI, 0.346–0.553, *P* = 0.014), heart failure (OR = 0.607, 95% CI, 0.465–0.792, *P* < 0.001), hypertension (OR = 0.745, 95% CI, 0.616–0.900, *P* = 0.002), migraine (OR = 0.320, 95% CI, 0.191–0.535, *P* < 0.001), total cholesterol (OR = 0.996, 95% CI, 0.994–0.998, *P* < 0.001), HDL (OR = 0.960, 95% CI, 0.953–0.966, *P* < 0.001), heart rate (OR = 0.984, 95% CI, 0.979–0.988, *P* < 0.001), and NIHSS > 7 (OR = 0.709, 95% CI, 0.602–0.835, *P* < 0.001) were all significantly associated with females. Coronary artery disease (OR = 1.675, 95% CI, 1.407–1.994, *P* < 0.001), alcohol use (OR = 3.960, 95% CI, 2.664–5.887, *P* < 0.001), smoking (OR = 1.442, 95% CI, 1.206–1.724, *P* < 0.001), serum creatinine (OR = 1.269, 95% CI, 1.146–1.405, *P* < 0.001), diastolic blood pressure (OR = 1.025, 95% CI, 1.021–1.030, *P* < 0.001), TPA administration (OR = 1.196, 95% CI, 1.007–1.421, *P* = 0.041), ambulation improvement (OR = 1.185, 95% CI, 1.010–1.391, *P* = 0.037), and sleep apnea (OR = 2.825, 95% CI, 1.810–4.409, *P* < 0.001) were all associated with the males. Our model indicates a strong discriminative capability (Area Under the Curve (AUC) = 0.758, 95% CI, 0.742–0.775, *P* < 0.001).

**Fig. 2:**
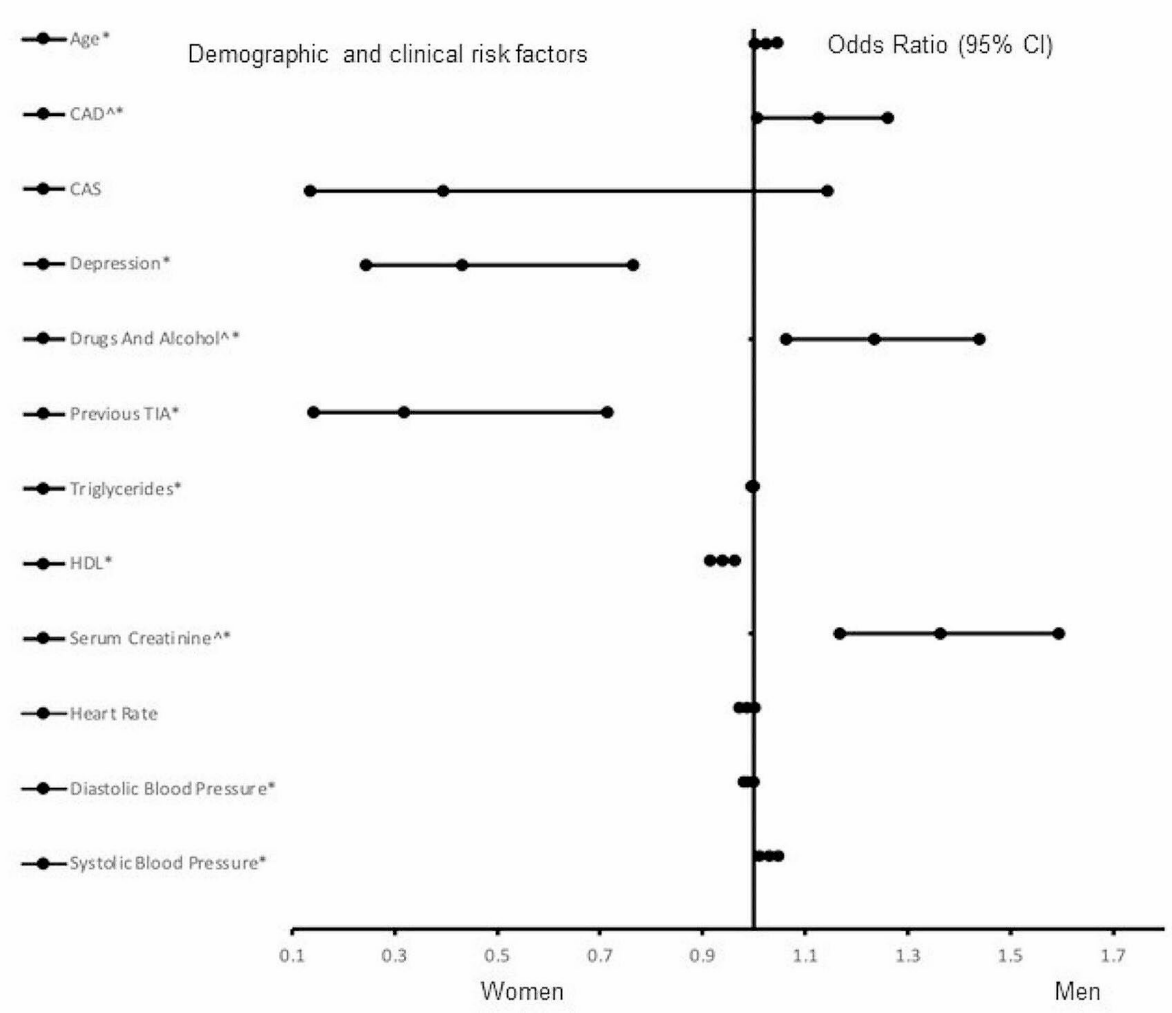
Risk factors associated with obese AIS population with a history of smoking. Odd ratios (OD) below 1 denotes factors associated with female while above 1 denotes factors associated with male patients. *Indicates statistical significance (*P* < 0.05) with a 95% confidence interval

Figure 2 presents risk factors for obese AIS population with a history of smoking. Coronary artery disease (OR = 1.806, 95% CI, 1.028–3.174, *P* = 0.040), a history of alcohol use (OR = 2.873, 95% CI, 1.349–6.166, *P* = 0.006), serum creatinine (OR = 4.724, 95% CI, 2.171–10.281, *P <* 0.001), age (OR = 1.024, 95% CI, 1.022–1.047, *P =* 0.033), and systolic blood pressure (OR = 1.029, 95% CI, 1.011–1.047, *P <* 0.002) were significantly associated with males. Depression (OR = 0.432, 95% CI, 0.244–0.764, *P =* 0.004), previous TIA (OR = 0.319, 95% CI, 0.142–0.714, *P <* 0.005), and higher levels of HDL (OR = 0.938, 95% CI, 0.915–0.962, *P <* 0.001) were associated with females. The discriminative capability of the model was strong (AUC = 0.769, 95% CI, 0.729–0.809, *P* < 0.001).

**Fig. 3:**
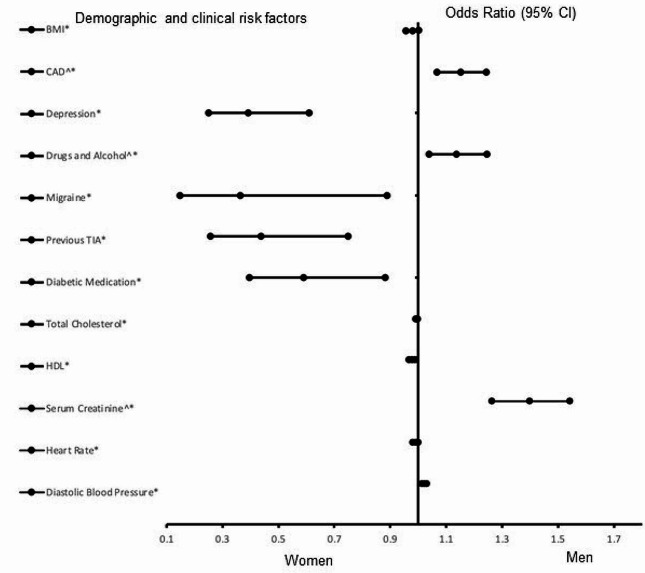
Risk factors associated with AIS patients with a history of smoking only. Odd ratios (OD) below 1 denotes factors associated with female while above 1 denotes factors associated with male patients. *Indicates statistical significance (*P* < 0.05) with a 95% confidence interval

Figure 3 presents risk factors for AIS patients with a history of smoking only. Coronary artery disease (OR = 2.033, 95% CI, 1.385–2.984, *P* < 0.001), a history of alcohol (OR = 1.906, 95% CI, 1.207–3.007, *P* = 0.006), serum creatinine (OR = 5.325, 95% CI, 3.225–8.711, *P <* 0.001), and diastolic blood pressure (OR = 1.022, 95% CI, 1.013–1.031, *P <* 0.001) were associated with males. Depression (OR = 0.391, 95% CI, 0.251–0.610, *P <* 0.001), previous TIA (OR = 0.0.438, 95% CI, 0.256–0.749, *P* = 0.003), diabetic medication (OR = 0.591, 95% CI, 0.396–0.882, *P* = 0.010), total cholesterol (OR = 0.995, 95% CI, 0.991–0.998, *P* = 0.002), HDL (OR = 0.978, 95% CI, 0.967–0.989, *P* = 0.002), and heart rate (OR = 0.990, 95% CI, 0.980–0.999, *P* = 0.025) were associated with females. The discriminative capability of the model was strong (AUC = 0.754, 95% CI, 0.727–0.781, *P* < 0.001).

**Fig. 4:**
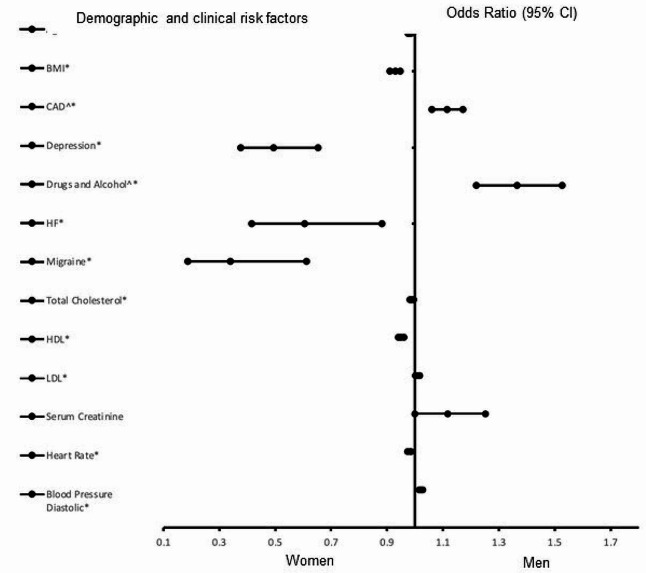
Risk factors associated with obese AIS patients only. Odd ratios (OD) below 1 denotes factors associated with female while above 1 denotes factors associated with male patients. *Indicates statistical significance (*P* < 0.05) with a 95% confidence interval

Figure 4 presents the risk factors of obese AIS patients. Coronary artery disease (OR = 1.720, 95% CI, 1.335–2.216, *P* < 0.001), a history of alcohol (OR = 4.726, 95% CI, 2.701–8.268, *P* < 0.001), LDL (OR = 1.009, 95% CI, 1.001–1.017, *P =* 0.030), and diastolic blood pressure (OR = 1.022, 95% CI, 1.015–1.028, *P <* 0.001) were associated with males. Age (OR = 0.985, 95% CI, 0.976–0.993, *P <* 0.001), BMI (OR = 0.929, 95% CI, 0.910–0.948, *P* < 0.001), depression (OR = 0.495, 95% CI, 0.376–0.653, *P* < 0.001) heart failure (OR = 0.606, 95% CI, 0.417–0.881, *P* = 0.009), migraine (OR = 0.339, 95% CI, 0.188–0.612, *P* < 0.001), total cholesterol (OR = 0.989, 95% CI, 0.983–0.996, *P* = 0.003), HDL (OR = 0.950, 95% CI, 0.940–0.961, *P* < 0.001), and heart rate (OR = 0.981, 95% CI, 0.974–0.987, *P* < 0.001) were associated with females. The model has a strong discriminative capability (AUC = 0.768, 95% CI, 0.747–0.788 *P* < 0.001).

In general, we observed differences and similarities (Table 3). In terms of similarities, for men, coronary artery disease and alcohol use were observed in obese AIS patients with a history of smoking. In contrast, an increase in serum creatine was only observed in obese AIS patients with a history of smoking. Moreover, an increase in diastolic blood pressure was observed among AIS patients with a history of smoking or obesity. In terms of differences, an increase in age was only observed in obese AIS patients with a history of smoking. In contrast, an elevated LDL was only observed in obese male AIS patients that present. In contrast, an increase in systolic blood pressure was only observed in obese AIS patients with a history of smoking. For females, depression was observed in obese AIS patients with a history of smoking. In addition, a previous history of TIA was observed in obese AIS patients with a history of smoking. Elevated HDL was observed in obese AIS patients with a history of smoking. An increase in heart rate and total cholesterol was observed among obese AIS patients with or without a history of smoking. In terms of differences, the use of diabetic medication was only observed in AIS patients with a history of smoking. In contrast, BMI, heart failure, and migraine were observed among obese female AIS patients.

**Table 3:** A summary of similarities and differences in risk factors

	Obesity + smoking	Smoking	Obesity
Men	Coronary artery disease, alcohol use, serum creatinine, age, systolic blood pressure.	Coronary artery disease, alcohol use, serum creatinine, diastolic blood pressure.	Coronary artery disease, alcohol use, diastolic blood pressure, LDL.
Women	Depression, previous TIA, HDL	Depression, previous TIA, diabetic medication, total cholesterol, heart rate	Depression, age, HDL, total cholesterol, heart rate, BMI, heart failure, migraine,

## Discussion

This study produced several results. First, in obese AIS patients with a history of smoking, that present with coronary artery disease, a history of alcohol use, higher serum creatinine and systolic blood pressure were more likely to be males. In contrast, depression, previous TIA, and higher levels of HDL were associated with females.

Second, among AIS patients that are current or previous smokers, but not obese, coronary artery disease, alcohol use, elevated serum creatinine, and diastolic blood pressure were associated with males, while depression, previous TIA, use of diabetic medication, elevated total cholesterol, and heart rate were associated with females.

Third, in obese AIS patients without a history of smoking, coronary artery disease, alcohol use, LDL, and diastolic blood pressure were associated with males, while older patients with higher BMI and a history of depression, heart failure, migraine, and elevated total cholesterol. HDL and heart rate were more likely to be females.

Our results for obese male AIS patients with a history of smoking have been reported by other studies for stroke patients with CAD [36, 37], alcohol use [38], serum creatinine [39], age [40], and systolic blood pressure [41]. Coronary artery disease (CAD) is indeed more prevalent in males than in females [42], and this has been attributed to several factors, including the protective effects of sex hormones such as estrogen in females [43]. Estrogen is known to have antioxidative and anti-inflammatory properties that can slow down the progression of atherosclerotic plaque, a key factor in the development of CAD [44]. This is significant because atherosclerosis is a key underlying process in the development of CAD. Estrogen’s influence on plasma lipids and lipoproteins is also beneficial, as it can lead to a more favorable lipid profile, further reducing the risk of atherosclerosis [45]. This protective effect may contribute to reducing the incidence of CAD in female obese AIS with a history of smoking compared with males.

We observed that obese male AIS patients that present with a history of smoking were more likely to be associated with a history of alcohol use. Males are more likely to use alcohol as a coping mechanism for stress, which can lead to higher rates of alcohol use and related problems [46]. Societal norms and expectations can influence drinking behaviors. For example, heavy drinking may be more socially acceptable for males, leading to higher rates of alcohol use and related problems. Therefore, compared with males, more females are lifetime abstainers, drink less, and are less likely to develop alcohol-related disorders [47], although females who drink in excess develop more clinical issues [48]. Moreover, males are more likely to be treated in emergency departments for alcohol use problems [49]. These findings support our current result in alcohol use being associated with obese male AIS patients with a history of smoking. In addition, different factors, including alcohol pharmacokinetics as well as its effect on nervous system functioning and the level of sex hormones, may interact with alcohol use in a complex manner [50], and contribute to the observed difference in male and female patients. It is important to point out that while these trends can be observed at the population level, individual experiences with alcohol use and its related problems can vary widely. Future studies on mechanisms underlying the biology and psycho-sociocultural differences in alcohol use, obesity, and smoking will help in the development of appropriate care for male and female obese AIS patients with a history of smoking.

Elevated serum creatine level was linked with obese male AIS patients with a history of smoking. An association between increased risk of cerebrovascular disease and elevated serum creatine levels has been reported [51, 52]. These studies focused on specific groups, such as patients with stroke [53], and myocardial infarction [53], in all of whom cerebrovascular and coronary heart disease are the primary causes of mortality. The authors suggested that an increased level of creatinine has a major impact on vascular diseases [54], implying that serum creatinine predicts survival in patients with stroke [55]. In our current study, elevated serum creatine was associated with AIS males who are obese and present with a history of smoking. This result indicates the relationship between serum creatinine and stroke events in AIS male patients who are obese and present with a history of smoking. Our finding indicates that an elevated serum creatinine concentration may represent a marker for subtle renal damage and may constitute an additional risk factor for the combined effect of obesity and smoking on male stroke patients. Therefore, monitoring serum creatinine concentration is crucial, especially for male stroke patients with a history of smoking and obesity.

Elevated systolic blood pressure was linked with male AIS patients who present with a history of smoking, while diastolic blood pressure was linked with obese AIS patients with or without a history of smoking, but not in patients who presented with the combined effect of obesity and smoking. Hypertension guidelines include both systolic and diastolic blood pressure (DBP) targets [56, 57]. It has been shown that although systolic blood pressure (SBP) had a greater effect, systolic and diastolic blood pressures each independently affect stroke outcomes [58]. Accordingly, elevated levels of SBP and DBP were associated with an increased risk of ischemic stroke in both sexes [59]. In general, the link between BP and severity of stroke is controversial [60, 61]. While some studies show that neither SBP nor DBP projected stroke severity [62], others suggest that only SBP [63] or DBP [64] were associated with worse neurologic outcomes. Our current study indicates that higher SBP was associated with male, obese AIS patients and present with a history of smoking, while DBP was associated with obese AIS patients with or without a history of smoking. Therefore, future studies may help determine the relationship between SBP, DBP, and obese AIS patients with a history of smoking.

We observed that LDL was only linked with obese male AIS patients, and not with AIS patients with the combined effect of smoking and obesity or smoking alone. Stroke is a multifactorial disease and is associated with several risk factors including atherosclerosis of the cerebral circulation, linked with the abnormalities of serum lipids and lipoproteins [65, 66]. Such aberrations include an increase in triglyceride levels, downregulation of high-density lipoproteins (HDL) cholesterol concentrations, and elevated low-density lipoproteins (LDL) levels [67]. In particular, LDL consists of many distinct subcomponents including LDL-1 to LDL-7 LDL, of which LDL-3 and LDL-4 are known risk factors for AIS [68]. Our finding that LDL was associated with male obese AIS patients underscores the importance of LDL and its sub-components in the assessment and management of these patients. Therefore, monitoring and managing these lipid levels is crucial in the prevention and treatment of stroke, particularly in male obese AIS patients.

An important finding in this study is that among patients with AIS, those who are obese and present with a history of smoking, either individually or in combination are more likely to experience depression. Depression is known to elevate morbidity in ischemic stroke patients [69]. Regardless of age, females exhibit higher rates of depression than males [70]. Females are more likely to be treated with depression even during stroke when compared with males [71]. Furthermore, females tend to experience poorer functional outcomes after ischemic stroke [72]. Therefore, given the higher rates of depression and worse outcomes after stroke in females, it is not surprising that female AIS patients who are obese and present with a history of smoking or obesity were associated with depression in our current study. This finding underscores the need for targeted interventions to manage depression, especially in female stroke patients and those with risk factors like obesity and smoking.

We observed that female obese AIS patients that present with or without a history of smoking were more likely to be associated with a baseline previous TIA. A previous history of TIA is a major predictor of subsequent ischemic stroke [73]. In general, the incidence of TIA varies and is stratified by age distribution. For example, among males between the ages of 65 to 69, TIA is lower (< 2.6%) when compared with those between 75 and 79 years of age(> 3.5%) [74]. For females that are between 65 and 69 years of age, the prevalence of TIA is 1.6%, while females between 75 and 79 years of age presented with a higher proportion (> 4.0%) [75]. In general, a higher prevalence of TIA is reported in females when compared with males [75] and this supports our current results in female AIS patients with a combined effect of obesity and smoking, and smoking alone. Future studies that focus on the management of TIA and related risk factors by sex are necessary to reduce TIA incidence in male and female patients.

We observed an association between elevated total cholesterol (TC) and female AIS patients with a history of smoking while higher levels of HDL were associated with obese AIS patients with a history of smoking. TC has been identified as an independent predictor of poor outcomes in patients with AIS as documented by several studies [76–78]. Furthermore, there is a significant association between sex and serum levels of TC, LDL-C, and HDL-C in stroke patients [79, 80]. Findings indicate that females with AIS present with higher serum levels of all three subclasses, including TC and HDL, compared to males [81], suggesting a sex-dependent relationship between TC and HDL serum levels. Moreover, females with higher TC present with an increased risk for AIS compared to females whose cholesterol is lower [82]. The current study indicates a higher level of HDL-cholesterol and LDL-cholesterol in obese female AIS patients with baseline history of smoking. This result highlights the complex interplay between cholesterol levels, obesity, smoking history, and sex in the context of AIS.

An increase in heart rate was associated with female AIS patients with a history of smoking or obesity, while an increase in heart failure (HF) was associated with obese female AIS patients. An increase in heart rate is a biological variable that can predict heart failure and cardiovascular diseases [83]. This suggests a link between increased heart rate and HF, which can be extended to vascular diseases, including stroke [84]. Heart failure manifests differently in males and females, and females are at a higher risk than males [85]. While ischemia is the primary cause in males, hypertension and diabetes contribute more to HF in females [85]. Our current finding indicates that an increase in HF was associated with female AIS patients with a history of smoking, or obesity. Further investigation into the pathophysiology and management approaches for individuals with a history of ischemic stroke considering males and females and taking into account their history of smoking, obesity, and baseline elevation in heart rate and heart failure will represent an advancement towards precision medicine and improvement of clinical research.

We observed that female AIS patients, that present with a history of smoking were more likely to be treated with diabetic medication. Diabetes significantly increases the risk of ischemic stroke, and this risk is higher in females compared to males [86]. Uncontrolled diabetes can lead to hyperglycemia, which can result in various outcomes, including mild, moderate, or severe intracerebral hemorrhage [87]. Notably, diabetes shows a stronger association with ischemic stroke in females compared with males [88], and the risk starts at lower fasting blood glucose levels for females [89]. AIS patients with diabetes that receive continuous diabetic medications both before stroke onset and after admission have improved functional outcomes [90]. This finding supports our current result highlighting the use of diabetes medication among female AIS, especially considering the stronger association between incident ischemic stroke in females compared with males [88].

There was an association between higher BMI and female AIS patients. Higher BMI or obesity is linked to a higher risk of ischemic stroke [91]. This positive association between higher BMI and ischemic stroke incidence has been reported in both males and females [92]. Although higher BMI is linked to a higher risk of ischemic stroke in both sexes, the association is notably stronger in females when compared to males [92]. This finding strengthens our current result indicating that female AIS patients are more likely to have a higher BMI.

In our study, we found that female AIS patients with a history of obesity were more likely to be associated with migraine. A similar trend has been reported for stroke patients [93]. Migraine is associated with an increased risk of ischemic stroke among males and females, but females are particularly more affected by migraine, especially considering that the condition is predominantly diagnosed in females [94]. The sex-related differences in migraine also carry clinical significance, as the incidence, duration, and disability associated with migraine tend to be higher in females [95]. Taken together, existing studies support our current result regarding the association of obese female AIS patients with baseline migraine.

An important finding in this study is that older male AIS patients were associated with male and female AIS patients with a combined effect of obesity and smoking, while older female AIS patients were more likely to be associated with patients with a current or previous history of smoking. Aging is a major risk factor for stroke and the risk increases every 10 years after age 55 years [96]. More than half of ischemic strokes occur in individuals who are ≥ 65 years old, and stroke-related mortality increases with age [96]. The death rates from stroke are expected to increase in people aged ≥ 65 years in the next 10 years [97]. Moreover, the general lifetime risk for stroke in males is proposed to be 1 in 6 and 1 in 5 for females [98], with males presenting with greater rates at young age and females at older ages. In our current study, the proportion of AIS with a history of smoking or obesity who are older (e.g. ≥80) is small compared with younger age groups, indicating that age, when combined with two or more chronic conditions such as obesity and smoking, are important risk factors in male and female stroke patients. Therefore, smoking and obesity either alone or combined, may potentially interact with conventional cardiovascular risk factor(s) to increase the risk, severity, and outcome in male and female AIS patients.

### Limitations

This study has many notable limitations. Data for smoking was obtained from self-report. While there is evidence supporting the validity of self-reported smoking [99], there is a tendency of bias because of under- or overreporting of smoking behaviors. Additionally, factors such as quantity, duration, and continuation of smoking habits were not included and could potentially have an impact on the results. Another limitation is that all patient data on clinical characteristics, risk factors, comorbidities, etc. were collected from a single institution and the results of this study cannot be generalized to other institutions. Moreover, our data is cross-sectional thus, limiting causal inferences. The retrospective nature of our study did not separate current and previous smokers in the database of the stroke registry, making it difficult to identify those who are current or previous smokers. This study has notable strengths. A major strength is the ability to determine risk factors that are associated with the combined effect of obesity with a current or previous history of smoking in male and female AIS patients. Our findings have implications for the future development of precise medicine for the care of male and female AIS patients with the combined effect of obesity with a current or previous history of smoking.

## Conclusion

Despite significant advancements in research focusing on sex differences in comorbidities associated with stroke, as well as the identification of specific risk factors influencing treatment outcomes in both female and male AIS patients, there are still notable gaps in research. Existing data on stroke often fail to distinguish between sex and gender integrating both influences without recognizing that sex and gender are not binary concepts. Experimental designs and data collection do not differentiate between sex and gender identity. Consequently, many findings on sex differences reflect a combined effect of both sex and gender [100] supporting the observed differences between male and female AIS patients in our current study. Our findings showed differences and similarities in risk factors for male and female patients. Precisely, we identified specific risk factors unique to male and female patients that when managed could enhance the care of AIS patients with a combined effect of obesity with a current or previous history of smoking. These findings highlight the need for the development of management strategies targeting AIS patients with a combined effect of obesity and smoking or smoking.

## Data Availability

Materials are available on request from the corresponding author.

## Abbreviations

AIS: Acute ischemic stroke
BMI: Body mass index
AUROC: Area under the receiver operating curve
aPTT: Active partial thromboplastin time
BBB: Blood-brain barrier
CVD: Cardiovascular disease
CAD: Coronary artery disease
CAS: Carotid artery disease
CRD: Chronic renal disease
CT: Computer tomography
INR: International normalized ratio
MRI: Magnetic resonance image
TIA: Trans ischemic attack
rtPA: Recombinant tissue plasminogen activator
OD: Odd ratio
HDL-C: High-density lipoprotein
LDL-C: Low-density lipoprotein
TC: Total cholesterol
TGs: Total triglycerides
NIHSS: National Institute of Health stroke scale
PVD: Peripheral vascular disease
rtPA: Recombinant tissue plasminogen activator
ROC: Receiver Operating Curve
sICH: Symptomatic intracerebral hemorrhage
SD: Standard deviation
TC: Total cholesterol
